# Generation of Trophoblast Stem Cells from Rabbit Embryonic Stem Cells with BMP4

**DOI:** 10.1371/journal.pone.0017124

**Published:** 2011-02-17

**Authors:** Tao Tan, Xianghui Tang, Jing Zhang, Yuyu Niu, Hongwei Chen, Bin Li, Qiang Wei, Weizhi Ji

**Affiliations:** 1 Kunming Primate Research Center, Kunming Institute of Zoology, Chinese Academy of Sciences, Kunming, Yunnan, China; 2 Graduate School of Chinese Academy of Sciences, Beijing, China; 3 Yunnan Key Laboratory of Animal Reproductive Biology, Kunming, Yunnan, China; 4 Kunming Biomed International, Kunming, Yunnan, China; Wellcome Trust Centre for Stem Cell Research, United Kingdom

## Abstract

Trophoblast stem (TS) cells are ideal models to investigate trophectoderm differentiation and placental development. Herein, we describe the derivation of rabbit trophoblast stem cells from embryonic stem (ES) cells. Rabbit ES cells generated in our laboratory were induced to differentiate in the presence of BMP4 and TS-like cell colonies were isolated and expanded. These cells expressed the molecular markers of mouse TS cells, were able to invade, give rise to derivatives of TS cells, and chimerize placental tissues when injected into blastocysts. The rabbit TS-like cells maintained self-renewal in culture medium with serum but without growth factors or feeder cells, whilst their proliferation and identity were compromised by inhibitors of FGFs and TGF-β receptors. Taken together, our study demonstrated the derivation of rabbit TS cells and suggested the essential roles of FGF and TGF-β signalings in maintenance of rabbit TS cell self-renewal.

## Introduction

In most mammals, the trophectoderm is one of the first cell types to be specified in the blastocyst. It surrounds the inner cell mass (ICM) and is responsible for the initiation of implantation. A subset of trophectoderm cells (trophoblast stem cells) retain the capacities to proliferate and to differentiate, eventually producing the entire trophoblastic population of the mature placenta, an ephemeral organ essential for nutrient and waste exchange between the fetus and its mother [1]. Trophectoderm differentiation and trophoblast formation are highly dynamic and finely regulated. Abnormalities in trophoblast formation and function underlie many aspects of early pregnancy loss and pregnancy complications in humans [2]. Experimentally modeling the in vivo process of trophoblast formation is difficult and presents a big challenge. However, trophoblast stem (TS) cells can be used to model and study the trophoblast in vitro [3].

Trophoblasts display morphological, functional and molecular diversity within and across species. Although some knowledge has been obtained from the study of mouse TS cells, which can be easily isolated from blastocysts, much less is known regarding human trophoblast development. To study the human trophoblast, several human trophoblast cell lines were derived from placental tissue or through immortalization of trophoblast cells [4], [5]. A recent study also reported the generation of cytotrophoblast stem cells from human ES cells [6]. These cells, however, failed to recapitulate the early stage of trophoblast development.

Embryonic stem (ES) cells and TS cells have distinct cell lineage fates and do not transdifferentiate in vivo or in vitro. However, recent studies demonstrated that genetic manipulation of the key players in trophoblastic lineage development, including forced repression of Oct4 [7] or over-expression of caudal-related homeobox 2 (Cdx2) or Eomes [8], can induce trophoblastic differentiation and permit the derivation of TS cells from ES cells. Moreover, ES cells cultured on embryonic feeder cells can be induced into trophoblastic differentiation by collagen IV or BMP4 [9], [10]. These studies indicated that ES cells have the ability to differentiate into trophoblastic lineage if they are provided with the correct clues.

Rabbit is a mating-induced ovulator. Its pregnancy can be precisely timed and the window of implantation can be readily defined by several biochemical markers [11], [12]. In addition, at the points where the blastocysts attach to the uterine epithelium, the trophectoderm forms unique structures known as trophoblastic knobs, which are readily identifiable during early pregnancy [13], [14]. For these reasons, rabbits and their TS cells appear to be ideal models to study the processes of implantation and placentation. We have established one rabbit ES cell line [15]. Using this ES cell line, we herein report the derivation of rabbit TS cells from ES cells differentiated with BMP4, which induced human ES cell differentiation into trophoblast [10]. We also provide evidences suggesting the essential roles of FGFs and TGFβ signalings in maintaining stem cell self-renewal. Rabbit ES cells and human ES cells display morphological and molecular similarities [15]. We therefore expected that rabbit TS cells would resemble human TS cells, and the knowledge obtained from studying rabbit TS cells could shed light on the process of human placentation.

## Results

### Derivation of epithelial-like cells in embryoid bodies (EBs)

**Figure 1:**
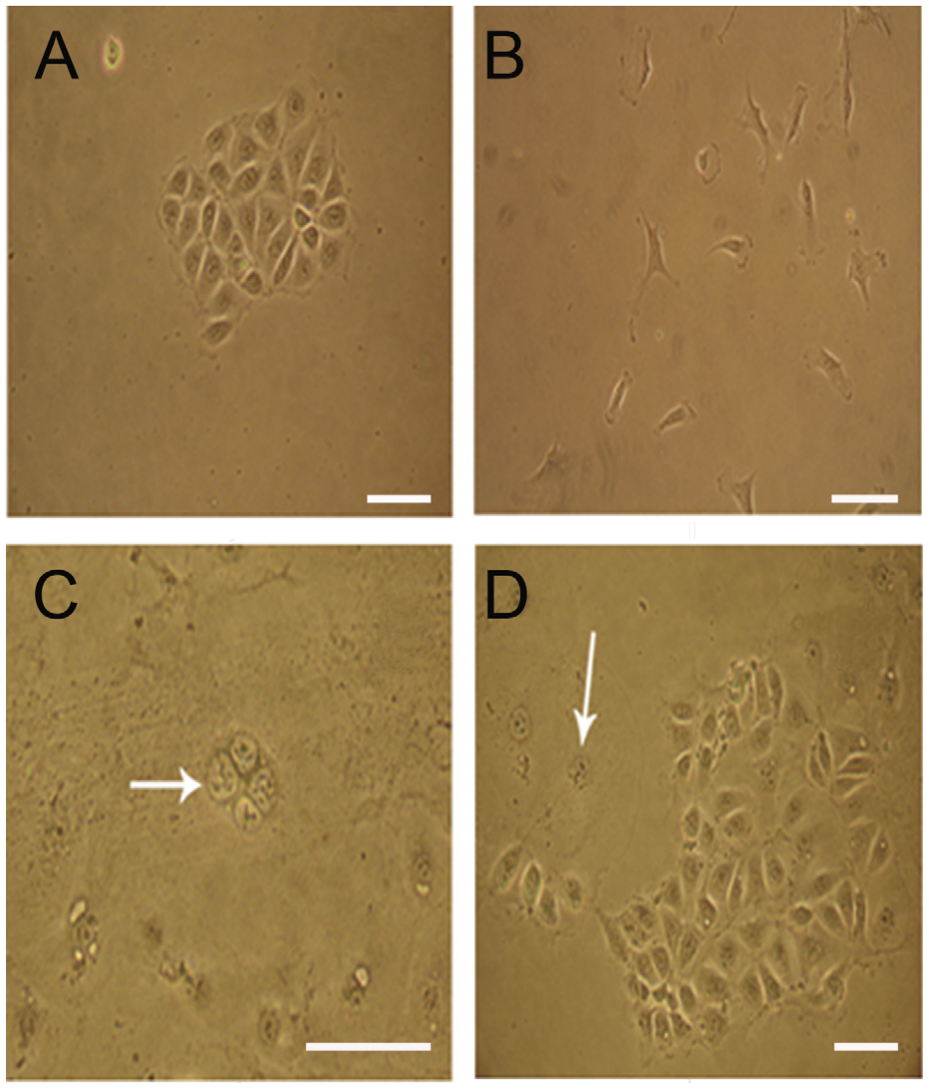
Derivation of epithelial-like cells from rabbit ES cells. (A) Epithelial-like cell clone. (B) Fibroblast-like cell clone. (C) Multinucleated cells formed in monolayer culture. Arrow pointed to the cell nucleus; magnification 200×. (D) Giant cells spontiniously differentiated from epithelial-like cell culture. Arrow pointed to cell nucleus. All magnifications were 100×, unless otherwise stated. The scale bar represents 100 µm.

Rabbit ES cells treated with BMP4 were induced to differentiate into epithelial-like cells in both EB and monolayer culture systems. In EB differentiation, cells displayed heterogeneity at the beginning of BMP4 treatment (day 0), with cuboidal epithelial-like cells surrounded by fibroblast-like cells at the edge. The epithelial-like cells proliferated faster than the fibroblast-like cells, leading to domination of the epithelial-like population at day 10–15 of differentiation. A few multinucleated cells were formed at this stage. There was no significant difference among the four groups of BMP4 treatment in term of differentiation rates (1, 5, 10 and 20 ng/ml). These epithelial-like (Figure 1A) and fibroblast-like cells (Figure 1B) were expanded via limited dilution and individual cell clones were established from single cells. The epithelial-like cells were capable of self-renewal and have been passaged up to 60 times. The doubling time was 16.084±0.379 hours.

In adherent differentiation, the epithelial-like cells appeared three days earlier in groups treated with high concentrations of BMP4 (10 or 20 ng/ml) when compared to those treated with lower concentrations (1 or 5 ng/ml) (3–4 days versus 6–7 days after treatment, respectively). Furthermore, the cells were homogeneous in morphology, without the formation of fibroblast-like cells (data not shown). However, these cells failed to sustain the morphological characteristics and eventually differentiated into multinucleated cells as well as giant nuclear cells (Figure 1C).

### Epithelial-like cells expressed genes characteristic of TS cells

**Figure 2:**
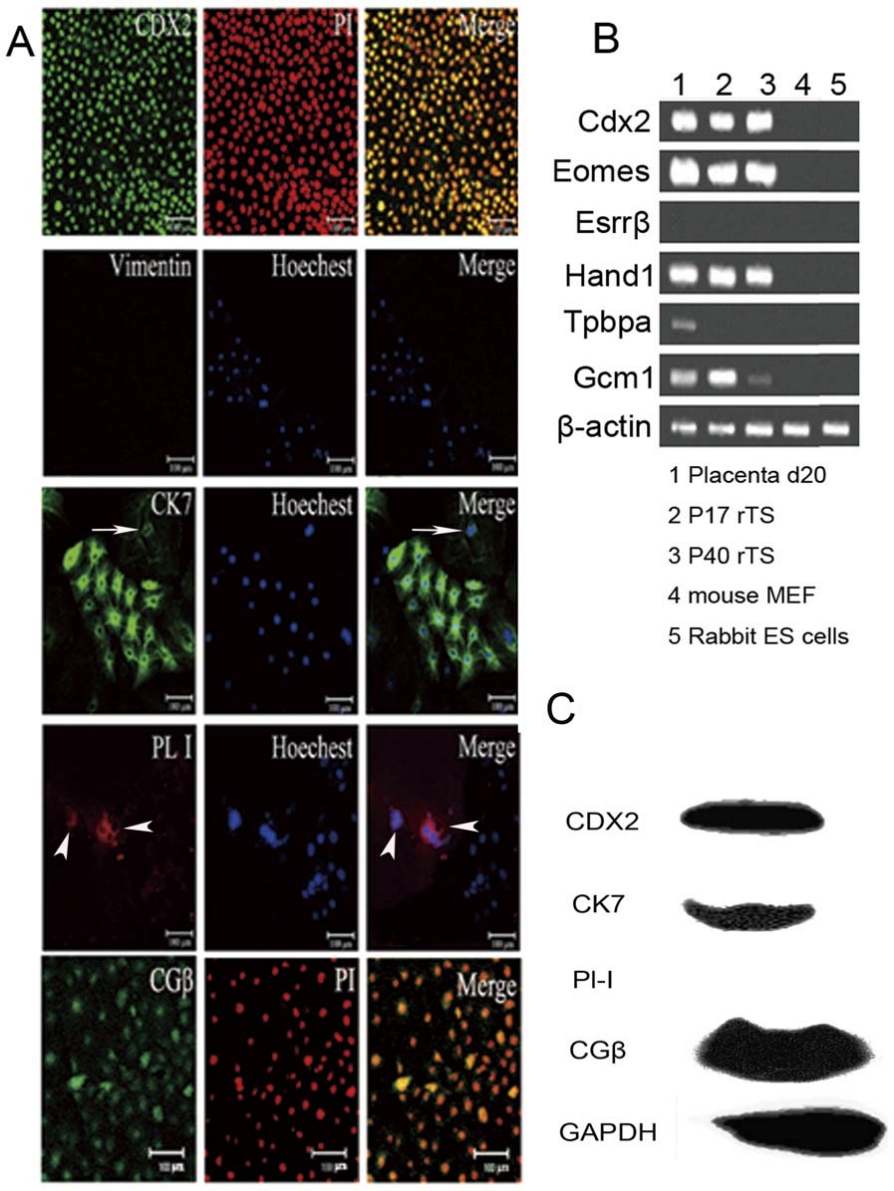
Epithelial-like cells expressed TS cell markers. (A) Immunofluorescence staining detected the ubiquitous expression of CDX2, CK7, and CGβ in epithelial-like cells. Note that the expression of CK7 by giant cells was weak (arrow), placental lactogen-1 (PL1) was only detected in giant cells (arrowhead), and Vimentin was absent in all cells. The scale bar represents 100 µm. (B) RT-PCR analysis of the expressions of TS cell markers in epithelial-like cells. Mouse MEF and rabbit ES cell cDNA were used as negative control and cDNA from d20 rabbit placenta was used as positive control. Lane 1, day 20 rabbit placenta sample; Lane 2, rTS cell sample at the 17th passage; Lane 3, rTS cell sample at the 40th passage; Lane 4, mouse embryonic fibroblast sample; Lane 5, rabbit ES cell sample. (C) Western blotting detected the expression of CDX2, CK7, placental lactogen-1 (PL1) and CGβ in rTS-like cells. The GAPDH expression was used as loading control.

The epithelial-like cells maintained self-renewal during continuous passages, although some cells spontaneously differentiated into giant nuclear cells (Figure 1D, arrow). These properties prompted us to examine if these cells are trophectodermal lineage stem cells. The mRNA expressions of Oct4, Nanog, Sox2 (three pluripotency genes), Cdx2, Esrrb, Eomes (three transcription factors characteristic of the trophectoderm in mouse and vole), Hand1 (known to promote the differentiation of giant trophoblast cells and is highly expressed in undifferentiated and differentiated mouse and vole TS cells), Gcm1 (syncytiotrophoblast marker) and Tpbpa (specific for spongiotrophoblast and ectoplacental cone) were examined by RT-PCR [7], [16], [17], [18]. As shown (Figure S1), the epithelial-like cells highly expressed the pluripotency marker Oct4 but not Nanog or Sox2 at passage 17. However, the expression level of Oct4 decreased dramatically at passage 40, as that of Gcm1 (Figure 2B), which might suggest stress [19]. The trophoblastic lineage markers Cdx2, Eomes, Hand1 and Gcm1 were consistently detected in epithelial-like cells at passages 17 (P17) and 40 (P40). Tpbpa mRNAs were expressed in rabbit placenta but not in epithelial-like cells. Surprisingly, the transcription factor Esrrb, which is expressed in TS cells and placenta of both mouse and vole [17], was not detected in either rabbit placenta or epithelia-like cells. Further studies were needed to verify the expression of Esrrb in rabbit. In consistency with the mRNA expression data, immunofluorescent staining and western blotting detected the expression of cytokeratin-7 (epithelial marker), CDX2, and chorionic gonadotropin β subunit (CG-β, trophoblast marker) [10] in these cells (Figure 2A, 2C). The germ layer markers Vimentin (Figure 2A), Nestin or Brachyury were not detected in these cells (data not shown). In accordance to the observation of spontaneous differentiation, Placental lactogen-I (PL-I), a specific marker for giant cells [20], was occasionally detected in some cells after prolonged culture by immunofluorescent staining (Figure 2A). Taken together, these data suggested that these epithelial-like cells were TS-like cells.

### TS-like cells can differentiate into trophoblastic derivatives in vitro and in vivo

**Figure 3:**
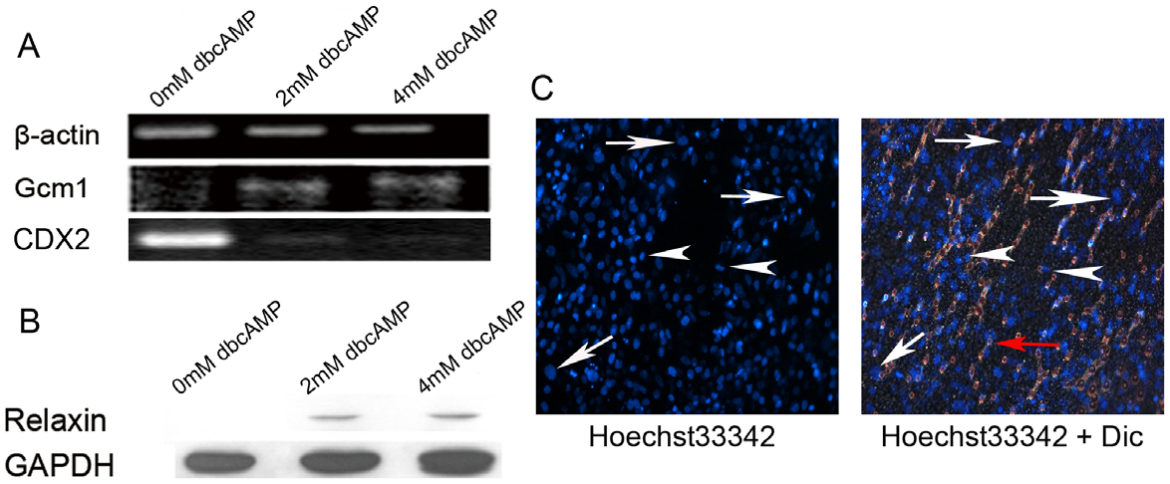
Rabbit TS-like cells can differentiate into trophoblastic derivatives *in vitro*. (A) Semi-quantitative RT-PCR analysis detected the increase in mRNA expression of Gcm1 (marker of syncytiotrophoblast) and the decrease of Cdx2 expression (trophoblast stem cell marker) in TS-like cells treated with different concentrations of dbcAMP. (B) Western blotting detected the expression of relaxin (syncytiotrophoblast marker) inTS-like cells treated with different concentrations of dbcAMP. (C) The TS-like cells cultured on Matrigel coated transwell invaded the transwell membrane. Fluorescent image (Hoechst 33342) and merged image of Hoechst 33342 with differential interference contrast (Dic) is shown. Arrows showed the giant nuclei of the penetrated cells and arrowheads showed the small nuclei of the cells. Red arrow indicated the pore of the transwell membrane. All analyses were repeated three times, and the representative figures are shown here.

To further clarify the identity of these TS-like cells, we went on to investigate if they have TS cell abilities to differentiate into trophoblast subtypes in vitro, and to chimerize placental tissues in vivo [21], [22], [23]. TS-like cells were treated with dibutyryl cAMP (dbcAMP) (0 mM, 2 mM and 4 mM) to induce differentiation into syncytiotrophoblast [24]. dbcAMP promoted transformation of TS-like cells into multinucleated cells in a concentration-independent manner. Similarly, time lapse microscopy revealed that adherent TS-like cells occasionally formed multinucleated syncytiotrophoblast when they met each other. Syncytiotrophoblasts were also formed through the fusion of mononucleated daughter cells (Movie S1). In accordance with the morphological change, the expression of the genes specific to the differentiated syncytiotrophoblast increased under the drug treatment. As shown, addition of dbcAMP increased Gcm1 expression and decreased CDX2 expression as detected by semi quantitative RT-PCR (Figure 3A). The protein level of relaxin, a marker of rabbit placental syncytiotrophoblast cells [24], was also elevated by dbcAMP treatment (Figure 3B). Moreover, the secretion of hormones (chorionic gonadotropin, progesterone and estradiol) by TS-like cells could be detected in the culture medium (Figure S2).

Invasion assay was utilized to examine if TS-like cells could differentiate into invasive trophoblastic giant cells. Matrigel-coated transwell mimic the three-dimensional structure of the endometrium, allowing assessment of the invasive capacity of trophoblast giant cells in vitro [25]. In the invasive assay of monolayer TS-like cells, an average of 406.7±33.0 cells (n = 3) had invaded the transwell to reach the bottom of the membrane. While most of the penetrating cells were characterized with giant nuclei (Figure 3C, arrows), some cells retained small nuclei (Figure 3C, arrow heads).

**Figure 4:**
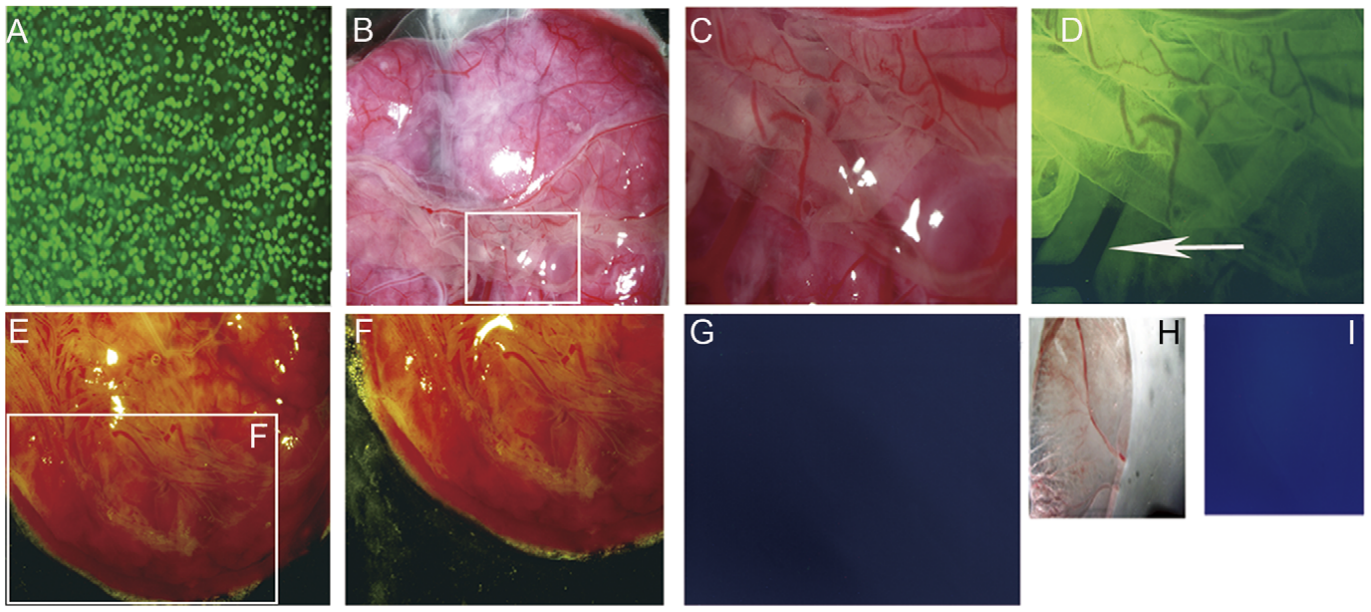
Rabbit TS-like cells can chimerize placentae *in vivo*. (A) GFP transgenic TS-like cells. (B) Whole-mount phase-contrast photographs of the undersides of chimeric placenta. White rectangle represents the part of placenta magnified in (C). (C) Magnification of part of the chimeric placenta in (B). (D) Fluorescent photograph of (C). The placenta showed an extensive contribution of GFP+ cells; Note that the blood vessels of the placenta had no fluorescence (arrow). (E) Whole-mount phase-contrast photographs of the undersides of control placenta developed from un-injected blastocyst. White rectangle represents the part of placenta magnified in (F). (F) Magnification of part of a non-chimeric placenta in (E). (G) Fluorescent photograph of (F). The control placenta showed weak background fluorescence. (H) Phase-contrast photograph of the Yolk sac from the chimeric embryo. (I) Fluorescent photograph of (H) Magnification of C, I, J is 4×, and F is 2×; Magnification of D, E is 7×, and G and H is 4×.

The above evidences prompted us to take the strictest test as if these TS-like cells were able to chimerize the placental tissues when injected into blastocysts. GFP transgenic TS-like cells (Figure 4A) were injected into blastocysts to examine their ability to chimerize the placental tissues. At day 20 of gestation, placenta proper, but not embryo proper of the conceptus (4 out of 12) derived from the green TS like-cell injected blastocysts emitted intensive green fluorescence (Figure 4B–D). In contrast, no green fluorescence was detected in placentas developed from uninjected control blastocysts (12 out of 12) (Figure 4E–G), demonstrating the chimerization of TS-like cells. Notably, we did not detect any fluorescence in placental blood vessels (Figure 4D, arrow) and the yolk sac (Figure 4H–I). Taken together, these evidences demonstrated the ability of rabbit TS-like cells to differentiate into trophoblastic derivatives in vitro and in vivo.

### FGF and TGFβ signalings are essential for TS-like cells self-renewal

**Figure 5:**
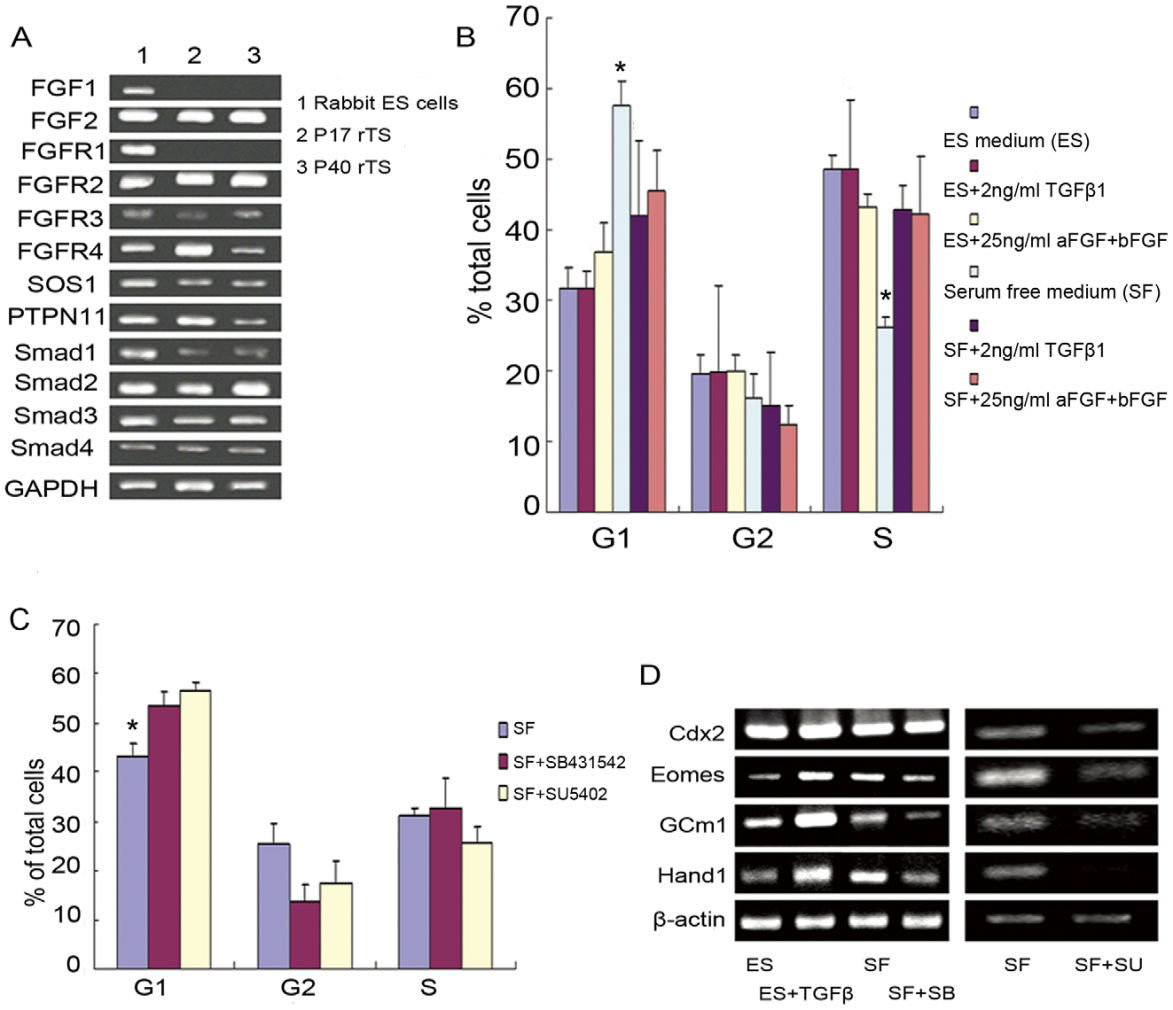
FGF and TGF-β signalings are essential for rabbit TS-like cells self-renewal. (A) RT-PCR detected the mRNA expressions of the key components of FGF and TGF-β signalings in TS-like cells. Lane 1, sample of rabbit ES cells; Lane 2, sample of TS-like cells at 17th passage; Lane 3, sample of TS-like cells at 40th passage. (B) FACS examination of cell cycle stages (G1, G2, and S phases) after TS-like cells were treated with growth factors for 48 hours in the presence or absence of serum. Note that in the absence of serum, growth factors stimulate cell proliferation (n = 3, * represents P<0.05). (C) FACS examination of cell cycle stages (G1, G2, and S phases) after TS-like cells were treated with growth factor inhibitors (20 µM of SB431542 against TGF-β type I receptor or 20 µM SU5402 against FGF receptor 1) for 48 hours in the serum-free medium (SF). Note that growth factor inhibitor treatments increased the percentages of TS-like cells at G1 phase (n = 3, * represents P<0.05). (D) Semi-quantitative RT-PCR analysis of mRNA expressions of the genes specific to trophoblastic lineage in TS-like cells cultured in ES medium (ES), ES medium with TGF-β (ES+TGF-β), serum free medium (SF), serum free medium with SB431542 (SF+SB), or serum free medium with SU5402 (SF+SU).

Previous studies reported the essential roles of FGF and TGF-β signaling in maintenance of mouse TS cell self-renewal [16], [26]. In this study, rabbit TS-like cells were propagated in culture medium supplemented with FBS but without growth factors under the feeder-cell free condition. To clarify if TS-like cell proliferation was regulated by FGF and/or TGFβ signaling, we cultured TS-like cells in the presence or absence of FBS supplied with or without these growth factors. Cell proliferation was monitored by the change of DNA content. As shown, the proliferation rate of TS-like cells was attenuated after withdrawal of FBS. The attenuation could be partially reversed by addition of 2 ng/ml TGF-β1 or 25 ng/ml aFGF+25 ng/ml bFGF into serum-free medium. However, addition of growth factors in the presence of FBS did not affect the proliferation rate (Figure 5B). These data suggested that FGF and TGF-β signalings were beneficial to TS-like cell proliferation, and their activation could be triggered by serum. To further confirm the indispensable roles of FGF and TGF-β signalings in the maintenance of TS-like cells, we treated the cells with FGF receptor 1 (FGFR1) inhibitor SU5402 or TGF-β type I receptor inhibitor SB431542 [27] to interrupt the signaling. In the absence of FBS, interfering either of the signaling pathways increased the population of cells at the G1 phase (Figure 5C), indicating that the proliferation of the TS-like cells was dependent of the two signalings. Concordantly, in TS-like cells we detected the mRNA expression of some components of FGF pathway (FGFR2, FGFR3, FGFR4, SOS1, PTPN11) and TGF-β pathway (Smad1, 2, 3, 4) (Figure 5A).

To test if FGFs and TGF-β signalings are able to maintain TS-like cell identity, the mRNA expression level of the genes specific to trophoblastic lineage (Cdx2, Eomes, Gcm1, and Hand1) was compared before and after 48 hour of treatment. As shown (Figure 5D), in the presence of FBS TGF-β1 had no effect on Cdx2 expression. However, it increased the mRNA expression of Gcm1, Hand1, and Eomes (another TS cell specific transcription factor downstream of Cdx2). In the absence of serum, interference of the TGF-β signaling with SB431542 led to the decreases in mRNA expressions of Eomes, Gcm1, and Hand1. Similarly, the reduction in mRNA expressions of Cdx2, Eomes, Gcm1, and Hand1 was observed when the FGF signaling was interrupted with SU5402 (Figure 5D). In contrast to the changes in gene expression level, the morphological transformation of TS-like cells was not obvious during 48 hour treatment. However, prolonged blockage of TGF-β or FGF signaling for more than four days caused the differentiation of TS-like cells into giant cells and syncytiotrophoblast followed by catastrophic death of all cell types (data not shown). Taken together, these evidences demonstrated the essential roles of these signalings in rabbit TS-like cells self-renewal.

## Discussion

Trophoblast stem cells are ideal models to study the biology of trophoblast [3]. In mouse, TS cells can be easily isolated from blastocysts. However, there has been no success in establishing human TS cells, albeit a study has reported derivation of stable, proliferating cytotrophoblast stem cells from human ES cells [6]. In this study, we have shown that rabbit ES cells were induced to differentiate into epithelial-like cells by BMP4. These epithelial-like cells expressed TS cell markers, were able to proliferate while maintaining their identity, to differentiate into trophoblastic derivatives *in vitro*, and notably, to chimerize the placental tissues *in vivo*. Thus, we demonstrated the establishment of rabbit TS cells from ES cells. Rabbit is phylogenetically closer to primates than mouse [28]. Our previous studies showed that rabbit and human ES cells, which had similar morphology, expressed the same stem cell markers (SSEA-3, SSEA-4, TRA-1-60 and TRA-1-81) and maintained self-renewal in the absence of LIF signal [15]. These similarities suggested that rabbit TS cell could be a suitable tool to study the molecular events occurring during human implantation and placentation.

Rabbit TS cells were derived from ES cells differentiated in EBs instead of in monolayer culture. Although differentiated cells in the monolayer culture system were more homogeneous in morphology than those derived in EBs, they lost proliferation capacity shortly after differentiation, as observed in human ES cells [10]. We speculated that EBs were able to provide the correct signals for ES cells to differentiate into TS cells. Otherwise, we could not exclude the possibility that gelatin, used in the monolayer culture, was not ideal for inducing differentiation of rabbit ES cells into TS cells.

The blastocyst is composed of two distinct cell lineages: inner cell mass (ICM) and trophectoderm (TE). ES cells are derived from ICM, while TS cells come from TE. ICM and TE, and ES cells and TS cells, correspondingly, have distinct lineage fates and do not transdifferentiate. ICM as well as ES cells contribute exclusively to the embryo proper, whilst TE or TS cells contribute to the extraembryonic tissues [29], [30], [31], [32], [33]. However, recent studies reported that ectopic manipulation of some genes or signaling pathways could divert ES cells from embryonic to trophoblastic fates. For instance, forced repression of Oct4 and Nanog or overexpression of Cdx2 and Hras could divert mouse ES cells toward trophoblastic fates [8], [34], [35], [36]. Collagen IV was also shown to induce mouse ES cells to differentiate into TS cells [9]. These reports altogether implicated ES cells as ideal models to investigate the molecular events governing the first cell fate determination during early embryogenesis. Rabbit ES cells were transdifferentiated into TS cells by treatment with BMP4, suggesting that BMP4 signaling plays key roles in the first cell lineage determination during rabbit embryogenesis. More studies are needed to confirm the existence and activation of BMP4 signaling in preimplantation embryos and to investigate its functions and the underlying molecular mechanisms in cell lineage determination.

The requirements for successful derivation and maintenance of TS cells vary across the species. For instance, propagation of mouse TS cells strictly requires MEF feeder cells (or feeder cell conditioned medium), FGF4 and TGF-β growth factors in the culture medium [16], [26]. Proliferation of vole and rat TS cells is FGF4 independent [17], [18]. In our study, rabbit TS cells do not require feeder cells to keep undifferentiated, but their self-renewal depends on the FGF and TGF-β signalings. TS cells of different species also express distinct stemness markers. Mouse and vole TS cells expressed Sox2 but not Oct4 or Nanog[17]. Rat TS cells expressed both Sox2 and Oct4[18]. Whereas rabbit TS cells expressed Oct4 but not Nanog or Sox2 in this study.

In summary, we have demonstrated the establishment and maintenance of rabbit TS cells. These knowledges could shed light on our understanding of human implantation and placentation.

## Materials and Methods

### Ethics Statement

This study was carried out in strict accordance with the recommendations in the Guide for the Care and Use of Laboratory Animals of the National research council. The protocol was approved by the Institutional Animal Care and Use Committee(s) (IACUC) of Kunming Institute of Zoology, Chinese Academy of Sciences (approval ID KIZ 20060011). All surgery was performed under isoflurane anesthesia, and all efforts were made to minimize suffering.

### Rabbit ES cell culture

Rabbit ES cells from one cell line derived in this laboratory were cultured as described [15]. Briefly, ES cells were seeded onto MEF feeders from E13.5 mouse fetuses (129/Sv) in gelatin and cultured in Dulbecco's modified Eagle's medium (DMEM, high glucose, without sodium pyruvate; Invitrogen, Carlsbad, CA, USA) supplemented with 2 mM glutamine, 0.1 mM mercaptoethanol, 1× Non-essential amino acids (Invitrogen), 1× penicillin-streptomycin and 15% defined fetal bovine serum (FBS; Hyclone, Logan, Utah) (referred to as ES medium thereafter). The cells were passaged with 5–10 mg/ml dispase every 3–5 days. All chemicals were from Sigma Chemical (St. Louis, MO, USA) unless otherwise stated.

### Derivation of rabbit TS-like cells from ES cells

Rabbit ES cells were differentiated in EB or in monolayers under the treatment of BMP4 [10]. In adherent differentiation ES cells were dispersed with 5 mg/ml dispase and plated onto gelatin-coated 6-well dishes (Becton Dickinson, Franklin Lakes, NJ, USA). Various concentrations of BMP4 (1, 5, 10 or 20 ng/ml; R&D Systems, Minneapolis, MN, USA) were included in ES medium for 7–15 days to promote differentiation. In EB differentiation, ES cells were digested with 5 mg/ml dispase, resuspended and cultured in hanging drops of ES medium for two days (30 µl/drop, 40 cells/µl). Aggregated EBs were then transferred to Petri dishes (Becton Dickinson) coated with agar to maintain continuous suspension cultures. After five or six days, the resultant cystic EBs were re-plated onto 6-well plates coated with 0.5% gelatin and cultured in the presence of 1, 5, 10 or 20 ng/ml BMP4 for 10–15 more days.

Limited dilution was utilized to isolate the epithelial-like cell colonies after differentiation. Briefly, cells were trypsinized, resuspended in ES medium and diluted to a density of 30 cells per 96-well plate, then plated onto a 0.5% gelatin coated 96-well plate (Becton Dickinson). Colonies were grown in ES medium and those exhibiting relatively homogeneous epithelium morphology were considered as promising epithelial-like cells [16] and picked for further expansion and downstream study. epithelial-like cells were expanded in ES medium until they reached confluence. They were replated at 1×10^3^/cm^2^ under the same culture conditions.

### *In vitro* differentiation of rabbit TS-like cells

To examine the differentiation potential of TS-like cells, dibutyryl cAMP (dbcAMP) was added to ES medium as described previously [24]. Briefly, 2.5×10^4^ cells/well were seeded onto a 6-well plate, and 2 or 4 mM dbcAMP was added to the culture medium. After treatment for 4 days, cells were harvested for analysis. All experiments were performed on cells that were between passages 20 to 30 unless otherwise stated.

### Immunofluorescence and Confocal Microscopy

Cells were fixed with 4% paraformaldehyde (PFA) for 10–15 min at 25°C and then rinsed three times in PBS, followed by permeabilization with 0.2% Triton X-100 for 10–15 min. Cells were then blocked in 5% goat serum for 30 min at 25°C and incubated with primary and secondary antibodies (Table S1) before imaging on a LSM 510 META confocal microscope (Carl Zeiss). Antibodies were obtained commercially and DNA was labeled with Hoechst 33342 or Propidium Iodide (PI). In each experiment, an isotype-matched IgG was used as negative control.

### Immunoblotting

As described [37], cells were washed and lysed in RIPA lysis buffer (Santa Cruz Biotechnology, Inc. Santa Cruz, CA, USA) for 1 hour on ice. Debris was removed by centrifuging at 12000×g for 15 min at 4°C. Equal amounts (25 µg) of samples were analyzed by SDS-PAGE. Immunoblots were performed with primary antibodies (Table S1) and horseradish peroxidase-conjugated secondary antibody (Table S1). Images were obtained with enhanced chemiluminescence (Pierce, Rockford, IL, USA), followed by exposure to Kodak autoradiography Biomax film (Kodak, Rochester, NY, USA). All experiments were repeated three times.

### RT-PCR and Semi-quantitative RT-PCR

Total RNA was extracted from rabbit placentas at day 20 of gestation (positive control), mouse MEF cells (negative control), rabbit ES cells, and clonal TS-like cells with Trizol (Invitrogen, Carlsbad, CA). RNAs were subjected to DNase I (Fermentas, Vilnius, Lithuania) treatment to remove possible genomic DNA contamination. Reverse transcription was carried out with approximately 2 µg of total RNA using RevertAid H Minus First strand cDNA synthesis kit (Fermentas, Vilnius, Lithuania). Due to the unavailability of the rabbit sequence information, specific primer sets (Table S2) were derived from the conserved sequences of the human, mouse and rat genes.

One µl of RT products was added to 1× Reaction Ready HotStart PCR master mix (Takara, Dalian, China) in 25 µl of final volume and amplified under the following conditions: 1 cycle at 95°C for 3 min; 25–35 cycles at 95°C for 30 sec, 56–60°C for 30 sec, and 72°C for 30 sec; and a full extension cycle at 72°C for 5 min. The PCR products were separated on 2% agarose gel and visualized after staining with ethidium bromide. For the semi-quantification RT-PCR, the intensity of the PCR band was semi-quantified with Quantity One quantitation software (Bio-Rad, Hercules, Calif). The relationship between the inverse of band intensity and the number of PCR cycles was linear.

### Invasion assay

Invasion assay was utilized to examine the invasive capacity of derivatives from TS-like cells [25]. Polycarbonate membrane transwell chambers with an 8 µm pore size (Corning, New York, US) were prepared for the invasion assay [25]. Membranes were coated with 1∶25 Matrigel (Becton Dickinson) in DMEM medium and rehydrated with DMEM medium the next day for 2 hours at 37°C under 95% humidity and 5% CO2. Suspensions of TS-like cells (6×10^4^ cells in 300 µl) were plated on top of the chambers in ES medium. Two days later, membranes were fixed in 4% PFA and the cells on top of each membrane were scraped off. The membrane was then stained with Hoechst 33342 before imaging on a LSM 510 META confocal microscope (Carl Zeiss). Five separate regions of the transwell membrane were randomly selected and the cells invading the membrane were counted manually.

### Growth factor or growth factor inhibitor treatments and DNA content analysis

To investigate the effects of growth factors on rabbit TS-like cell proliferation, cells were cultured with growth factors or growth factor inhibitors. In the growth factor treatments, 1×10^4^ cells were seeded as the starting point, and 1×10^5^ cells in the case of inhibitor treatments. Cells were seeded in 6-well plates in ES medium overnight followed by synchronization in serum-free (SF) medium for 24 hr. The cells were then cultured for 48 hour in the presence of growth factors or growth factor inhibitors. The growth factor treatments included: 1) control (SF medium); 2) 5 ng/ml recombinant TGF-β1 (Chemicon International Inc, Temecula, CA, USA); and 3) 25 ng/ml aFGF+25 ng/ml bFGF (Chemicon International). The inhibitor treatment groups were as followings: 1) control (SF medium); 2) inhibitor of TGF-β signaling, SB431542 (20 µM, Tocris Cookson Inc, Ellisville, USA) [27]; 3) FGF receptor inhibitor, SU5402 (20 µM, Tocris Cookson Inc, Ellisville, USA). Following treatment, cells were harvested and fixed in pre-cooled 70% ethanol at 4°C for 2 hour before incubation with 50 µg/ml propidium iodide plus 100 µg/ml RNase A in PBS for 30 min at 37°C. The DNA content was analyzed with FACS vantage SE (BD Biosciences), and the data were obtained using Cell Quest Software (BD Biosciences).

### Lentiviral infection of rabbit TS-like cells

TS-like cells were transfected with lentiviral vector expressing GFP. Briefly, Lentiviral vector FUGW constitutively expressing GFP (ubiquitin-C promoter–GFP) was generated as reported [38]. HEK-293T cells were co-transfected with GFP expression vector and viral packaging vectors pCMV-dR8.91 and pCMV-VSV-G using lipofectamine 2000 (Invitrogen). Lentiviral supernatants were collected 72 hour after transfection, and filtered through a 0.45 µm sterile filter. The viral particles were concentrated by ultracentrifugation at 100,000×g followed by reconstruction in PBS. Virus titre was determined immediately in HEK-293T cells (1×10^7^ virus/µl). Rabbit epithelial-like cells were cultured in 500 µl of ES medium containing 10 µl lentivirus and 5 µg/ml polybrene at 37°C for 48 hour. Thereafter, cells were trypsinized and resuspended for FACS sorting (Becton Dickinson). GFP-positive TS-like cells were collected, expanded, and subjected to one more FACS sorting in order to purify the transgenic cell population.

### Injection of rabbit GFP transgenic TS-like cells

Ten to twenty GFP transgenic TS-like cells in ES medium were injected into rabbit blastocysts. Uninjected blastocysts were used as negative control. The injected blastocysts were then transferred into uterine of pseudo-pregnant female rabbit [16]. Each time six control and TS-like cell injected blastocysts were transferred back into opposite uterine horns of each pseudo-pregnant female rabbit, respectively. Placentas and embryos at gestation day 20 were dissected and photographed on a Leica MZ16A microscope. The experiments were repeated two times. All animal experiments were approved by the Institute's research animal resource committee.

### Statistical analysis

The results were presented as means±standard error of the mean (SEM). The statistical analyses were performed using SPSS version 11.0 statistic software. The percentages of cell cycle distributions were transformed by arcsine of the square root prior to ANOVA analysis followed by Tukey's test. P≤ 0.05 indicated a significant difference.

## Supporting Information

Table S1 **Antibodies used in this research.**
(DOC)

Table S2 **PCR primers and conditions for gene expression analysis.**
(DOC)

Figure S1 **The mRNA expression of pluripotency genes (Oct4, Sox2, Nanog) in rabbit TS-like cells (rTS-like).** The mRNA expression of Oct4, but not Sox2 and Nanog was abundant in rTS-like cells at passage 17. The expression level decreased dramatically at passage 40.
(TIF)

Figure S2 **Secretion of placental hormones by rabbit TS-like cells.** The concentrations of CG (A), estradiol (B), and progesterone (C) in culture medium in the presence (+TS) or absence (-TS) of TS-like cells.
(TIF)

Movie S1 Time lapse microscopy revealed that adherent rabbit TS-like cells occasionally formed multinucleated syncytiotrophoblast when they met each other. Syncytiotrophoblasts were also generated through fusion of mononucleated daughter cells.
(MOV)
