# Supplementary material for: Generation of Trophoblast Stem Cells from Rabbit Embryonic Stem Cells with BMP4
Source: PLoS One. 2011 Feb 17;6(2):e17124. doi: 10.1371/journal.pone.0017124 (PMC3040765; doi:10.1371/journal.pone.0017124)
Supplement: Table S1 — Antibodies used in this research. (DOC) [file pone.0017124.s001.doc]

**Table S1. Antibodies used in this research**

| Antibody against | Antibody type | Dilution | Source |
| --- | --- | --- | --- |
| CDX2 | Mouse monoclonal antibody IgG1 | 1: 200 | Abcam (Cambridge, MA) |
| Cytokeratin 7 (CK7) | Mouse monoclonal antibody IgG1 | 1: 200 | DAKO |
| Vimentin | Mouse monoclonal antibody IgG1 | 1: 200 | DAKO |
| Placental lactogen-Ⅰ | Mouse monoclonal antibody IgG1 | 1:100 | Abcam (Cambridge, MA) |
| CGβ | Mouse monoclonal antibody IgG1 | 1:200 | DAKO |
| Fluorescein (FITC)-conjugated goat anti-mouse IgG1 | Goat polyclonal  antibody IgG | 1:200 | Santa Cruz Biotechnologies |
| Texas Red (TR)-conjugated goat anti-mouse IgG1 | Goat polyclonal  antibody IgG | 1:100 | Santa Cruz Biotechnologies |
| glyceraldehyde-3-phosphate dehydrogenase (GAPDH) | Mouse monoclonal antibody IgG1 | 1:5000 | KangChen Bio-tech Inc.  (Shanghai China) |
| Relaxin | Goat polyclonal antibody IgG | 1:1000 | Santa Cruz Biotechnologies |
| horseradish peroxidase (HRP )- conjugated donkey anti-goat IgG | Donkey polyclonal antibody IgG | 1:5000 | Abcam (Cambridge, MA) |
| horseradish peroxidase (HRP )- conjugated goat anti-mouse IgG | Goat polyclonal antibody IgG | 1:5000 | Santa Cruz Biotechnologies |
