# Supplementary material for: Generation of Trophoblast Stem Cells from Rabbit Embryonic Stem Cells with BMP4
Source: PLoS One. 2011 Feb 17;6(2):e17124. doi: 10.1371/journal.pone.0017124 (PMC3040765; doi:10.1371/journal.pone.0017124)
Supplement: Table S2 — PCR primers and conditions for gene expression analysis. (DOC) [file pone.0017124.s002.doc]

**Table S2. PCR primers and conditions for gene expression analysis**

| Genes | Primer sequences  (5'-3', Forward and Reverse) | Annealing temp | Length (bp) | Accession No. |
| --- | --- | --- | --- | --- |
| Cdx2 | 5'ACCTGTGCGAGTGGATGC3' 5'CTTGGCTCTGCGGTTCTG3' | 56 | 230 | NM_007673 |
| Eomes | 5'ACTCAATCCCACCGCCCACTA3' 5'CACGCCATCCTCTGTCACTTCAACG3' | 56 | 313 | ENSOCUT00000000452 |
| Hand1 | 5'GGAGACGCACCGAAAGCATTA3' 5'GAGGAAAGCCTTCGTGTTGC3' | 56 | 255 | ENSOCUT00000011096 |
| Gcm1 | 5'TCCCAGAACTCTTCCGAACCCA3'  5'CATCATTTCTTCCTCAAACTCCCACT3' | 56 | 264 | ENSOCUT00000009245 |
| Tpbpa | 5'TGACCAGGAGACGGAAGG3' 5'GTTACTGTGGCTGATTTG3' | 56 | 206 | NM009411 |
| Esrrβ | 5'GACTCGCCGCCTATGTTC3' 5'CGATGTTGCCTTGAATGGT3' | 57 | 238 | NM_011934 |
| Oct4 | 5'AAGAGAAAGCGAACGAGTATTGAGA3'  5'TCCCAAAACTCCTTGCCCCACCCTT3' | 58 | 542 | NM_001099957.1 |
| Sox2 | 5'AAGAACAGCCCGGACCGCGTCAAGC3'  5'GCGTAGCTGTCCATGCGCTGGTTCA3' | 58 | 380 | XM_002716451.1 |
| Nanog | 5'GTGCCCGAACCTGAAGGGAAACATC3'  5'TTACATTTCATTCTTTGGTTCTGGA3' | 58 |  | XM_002712762.1 |
| FGF1 | 5'GGAGCGACCAGCACATTC3'  5'TCCCGTTCTTCTTGAGGC3' | 56 | 237 | NM_000800  NM_010197 |
| FGF2 | 5'GAAGAGCGACCCTCACATC3'  5'CCCAGTTCGTTTCAGTGCC3' | 59 | 232 | NM_002006  NM_008006 |
| FGFR1 | 5'AGATAACACCAAACCAAACCGTATG3'  5'GCATGCAATTTCTTTTCCATCTT3' | 56 | 330 | UniSTS:273319 |
| FGFR2 | 5'AAGGTACGAAACCAGCACTGGAG3' 5'TCCATCTCCGTCACATTGAACAG3' | 56 | 380 | UniSTS:464789 |
| FGFR3 | 5'CGGAAAGTTCGTCGCTGG3' 5'TTACTGGGCCCTGAGTCTGG3' | 54 | 150 | UniSTS:34762 |
| FGFR4 | 5'GGTGCAGACATGAGCAAGG3' 5'CAAGAAGCCGAGCAGAACC3' | 54 | 270 | UniSTS:55376 |
| SOS1 | 5'CCACCTCAGGAGAACAAA3' 5'CTCATACGGGTCAAATGC3' | 53 | 308 | NM_005633  NM_009231 |
| PTPN11 | 5'AGAGCAACGACGGCAAGT3' 5'TCAAAGGGCAGGATGTTT3' | 53 | 382 | NM_002834  NM_011202 |
| Smad1 | 5'GTTCAGGCAGTTGCTTACGA3' 5'AGGCATTCCGCATACACC3' | 55 | 266 | NM_001003688  NM_008539 |
| Smad2 | 5'CTTGATGGCCGTCTTCAGGT3' 5'GGGCGGCAGTTCTGTTAG3' | 57 | 246 | NM_001003652  NM_010754 |
| Smad3 | 5'TCAGAACCAGGACCAGAGCT3' 5'ACCACCACTTTCTCCCTGTG3' | 60 | 258 | UniSTS:64804 |
| Smad4 | 5'AGGTGGCTGGTCGGAAAG3' 5'TTGGCGGGTGTTGGATGGT3' | 57 | 312 | NM_005359  NM_008540 |
| β-actin | 5'CTA CAA TGA GCT GCG TGT GG3'  5'TAG CTC TTC TCC AGG GAG GA3' | 60 | 450 | NM_001101 |
| GAPDH | 5'TGAAGGTCGGAGTCAACGGA3'  5'TGGTGCAGGAGGCATTGCTG3' | 60 | 451 | NM_002046 |
