# Supplementary figures and images for: Generation of Trophoblast Stem Cells from Rabbit Embryonic Stem Cells with BMP4
Source: PLoS One. 2011 Feb 17;6(2):e17124. doi: 10.1371/journal.pone.0017124 (PMC3040765; doi:10.1371/journal.pone.0017124)

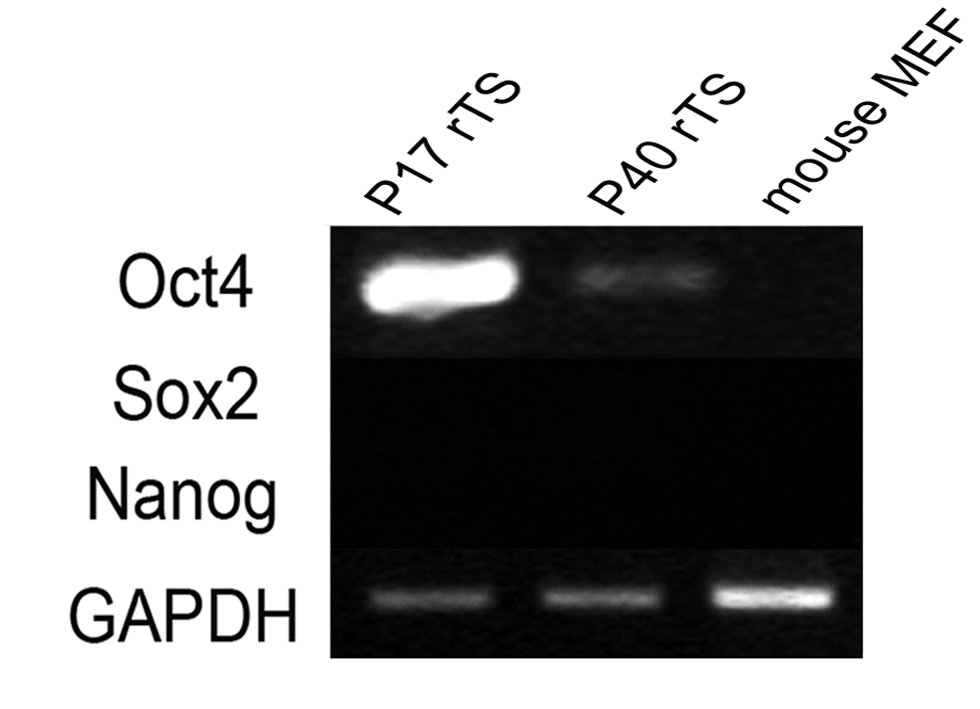

Supplement: Figure S1 — The mRNA expression of pluripotency genes (Oct4, Sox2, Nanog) in rabbit TS-like cells (rTS-like). The mRNA expression of Oct4, but not Sox2 and Nanog was abundant in rTS-like cells at passage 17. The expression level decreased dramatically at passage 40. (TIF) [file pone.0017124.s003.tif]

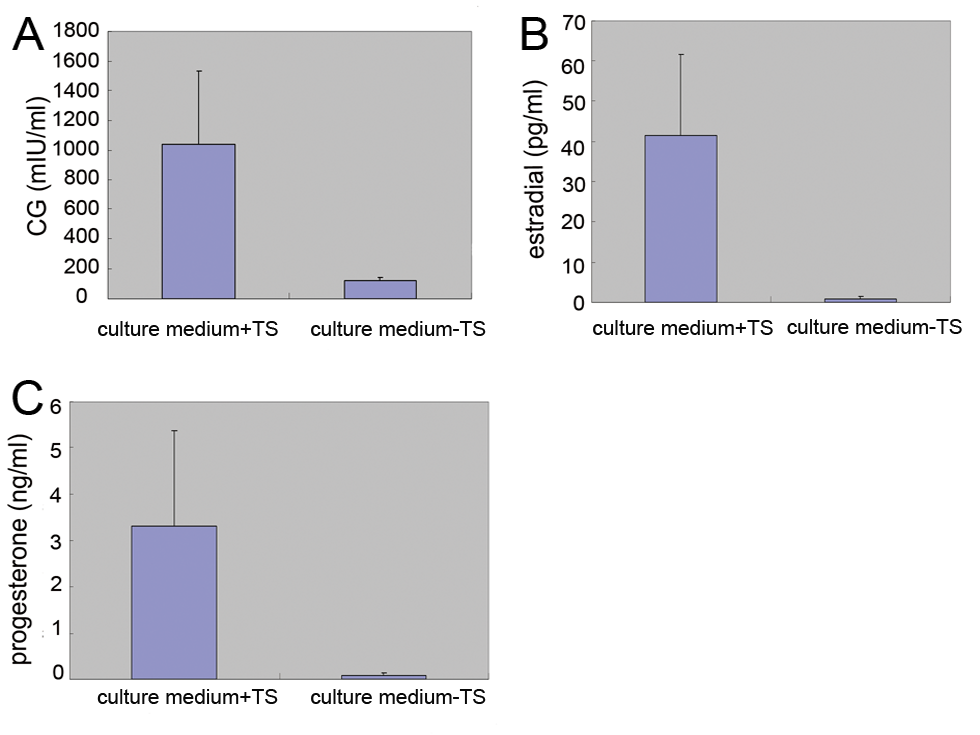

Supplement: Figure S2 — Secretion of placental hormones by rabbit TS-like cells. The concentrations of CG (A), estradiol (B), and progesterone (C) in culture medium in the presence (+TS) or absence (-TS) of TS-like cells. (TIF) [file pone.0017124.s004.tif]
